# Supplementary material for: Transcriptomic Insights into Mechanisms of Early Seed Maturation in the Garden Pea (Pisum sativum L.)
Source: Cells. 2020 Mar 23;9(3):779. doi: 10.3390/cells9030779 (PMC7140803; doi:10.3390/cells9030779)

Zhewan−1(10) vs Zhew

Global Summary

%DE = 0.9

genes with fdr < 0.2 = 1911 (729 + / 1182 −)

genes with fdr < 0.1 = 1777 (663 + / 1114 −)

genes with fdr < 0.05 = 1715 (631 + / 1084 −)

genes with fdr < 0.01 = 1338 (469 + / 869 −)

genes in genesets = 0

<FC> = 0

<t−score> = 5338490.75

<p−value> = 0

<fdr> = 0.1

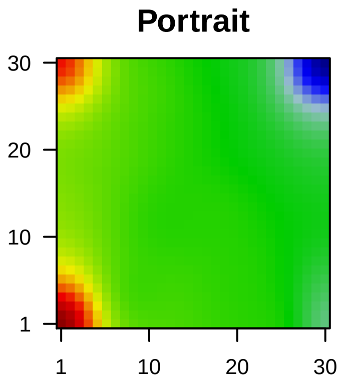

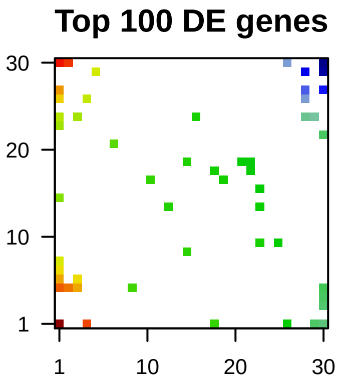

Global Genelist

Rank	ID	log(FC)	p−value	fdr	Metagene	Description
1	TRINITY_DN4	4.49	0e+00	7e−10	1 x 1	
2	comp40944_c	0	4e−12	1e−07	16 x 24	
3	TRINITY_DN2	0.03	8e−10	1e−07	11 x 17	
4	TRINITY_DN9	−2.89	9e−10	2e−07	30 x 30	
5	TRINITY_DN1	1	2e−09	2e−07	1 x 30	
6	TRINITY_DN5	3.25	3e−09	2e−07	1 x 30	
7	TRINITY_DN1	0.49	3e−09	3e−07	1 x 8	
8	TRINITY_DN1	−3.49	1e−08	3e−07	30 x 30	
9	TRINITY_DN1	9.38	1e−08	3e−07	1 x 1	
10	TRINITY_DN3	5.74	1e−08	3e−07	1 x 1	
11	TRINITY_DN2	−2.76	2e−08	3e−07	30 x 30	
12	TRINITY_DN2	−0.03	2e−08	3e−07	19 x 17	
13	TRINITY_DN1	17.12	2e−08	3e−07	1 x 1	
14	TRINITY_DN1	−2.9	2e−08	3e−07	30 x 30	
15	TRINITY_DN8	0.37	2e−08	3e−07	1 x 23	
16	TRINITY_DN1	0.29	2e−08	3e−07	1 x 24	
17	TRINITY_DN6	0.32	2e−08	3e−07	4 x 26	
18	TRINITY_DN2	2.12	2e−08	5e−07	1 x 1	
19	TRINITY_DN1	2.35	3e−08	5e−07	1 x 1	
20	TRINITY_DN1	−0.28	3e−08	5e−07	30 x 22	

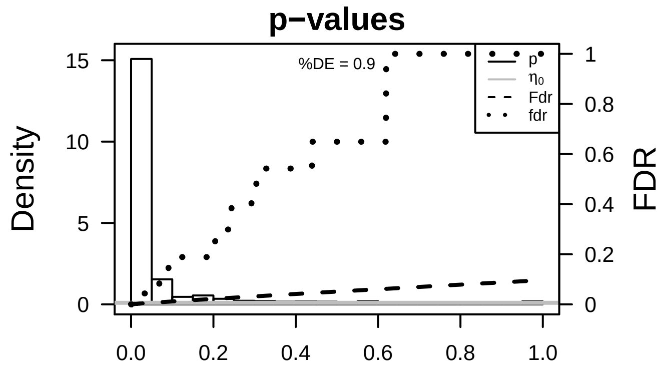

Supplement: Supplementary file 1 [file cells-09-00779-s001.zip › Supplementary materials/FileS4.zip!/FileS3/TFs - Results/Summary Sheets - Differences/Reports/Zhewan.1.10..vs.Zhewan.1.25..pdf]
